# Thromboelastography in COVID-19 patients: An observational study in the South African context

**DOI:** 10.4102/ajlm.v14i1.2681

**Published:** 2025-06-26

**Authors:** Bavinash Pillay, Sarah A. van Blydenstein, Shahed Omar

**Affiliations:** 1 Department of Internal Medicine, Faculty of Health Sciences, University of the Witwatersrand, Johannesburg, South Africa; 2 Department of Internal Medicine, Faculty of Health Sciences, Chris Hani Baragwanath Hospital, Johannesburg, South Africa; 3 School of Clinical Medicine, Department of Critical Care, Faculty of Health Sciences, University of the Witwatersrand, Johannesburg, South Africa; 4 Department of Critical Care, Faculty of Health Sciences, Chris Hani Baragwanath Hospital, Johannesburg, South Africa

**Keywords:** thromboelastogram, low molecular weight heparin, heparin, coronavirus disease 2019, viscoelastic methods, coagulopathy

## Abstract

**Background:**

Coronavirus disease 2019 (COVID-19) increases the risk of venous thromboembolism, requiring monitoring of low molecular weight heparin (LMWH) via a time-consuming, costly and often unavailable test – anti-factor Xa (anti-Xa). An affordable, rapid point-of-care alternative, the thromboelastogram, is available, but performance comparisons to anti-Xa are lacking.

**Objective:**

This study evaluated the relationship between anti-Xa and thromboelastogram in patients with COVID-19 receiving LMWH.

**Methods:**

This was a retrospective study of patients with COVID-19 receiving LMWH at Chris Hani Baragwanath Academic Hospital, Johannesburg, South Africa, between November 2020 and January 2021. Blood samples tested with thromboelastogram and anti-Xa were drawn at three timepoints (one prior to and two after administration of LMWH). Thromboelastogram parameters comprised reaction time (R-time; onset of testing to the start of clot formation), kinetics time (K-time; start of clot formation until the clot reached 20 mm), and thromboelastogram coagulation index (overall coagulation status of whole blood).

**Results:**

Forty-two patients with COVID-19 (15 male and 27 female) met the study criteria. There was a statistically significant, low to moderate correlation (Spearman’s correlation coefficient [*r*_s_ 0.43, *p* = 0.014]) between anti-Xa and thromboelastogram coagulation index. A statistically significant moderate correlation (*r*_s_ 0.52, *p* = 0.002) between anti-Xa and R-time, and a statistically significant low correlation (*r*_s_ 0.35, *p* = 0.049) between anti-Xa and K-time, were found. All correlations were 48 h post admission.

**Conclusion:**

Thromboelastogram coagulation index, R-times and K-times had a statistically significant association with anti-Xa levels in patients with COVID-19. Further research is required regarding their clinical utility.

**What this study adds:**

Thromboelastograms may represent a more cost-effective and accessible option to the conventional anti-Xa test in patients receiving LMWH. However, future research with larger sample sizes, varying disease profiles, and severity of illness is required.

## Introduction

Coronavirus disease 2019 (COVID-19)-infected patients are known to be at higher risk of venous thromboembolism (VTE).^1^ An accurate incidence rate of VTE in COVID-19-positive patients remains unknown, ranging from 4.8% to 85.0%.^2^ Autopsies of 12 patients with severe acute respiratory syndrome coronavirus 2 (SARS-CoV-2), revealed a VTE incidence of 58.0%.^3^ In an observational study of 150 patients admitted to the intensive care unit in France, pulmonary emboli occurred in 20.6% of patients.^3^ A cohort of 184 patients admitted to intensive care units in the Netherlands showed that pulmonary emboli occurred in 33.3% of all patients suffering from SARS-CoV-2 acute respiratory distress syndrome.^3^ The incidence of pulmonary embolism was found to be twice as high in patients with SARS-CoV-2 compared to patients with influenza.

The pathophysiology of VTE in COVID-19 is multifactorial. The infection is associated with an immune-mediated exaggerated inflammatory response, namely a cytokine storm including tumour necrosis factor-alpha, interleukin-8, and interleukin-6.^4^ Other contributory factors include endothelial injury, abnormalities in gas exchange and lung compliance, as well as haemodynamic changes.^2^

Owing to the high occurrence of VTE in patients with SARS-CoV-2, the International Society of Thrombosis and Haemostasis recommends that all clinicians should employ a high level of suspicion in the diagnostic work-up of deep venous thrombosis and pulmonary embolism.^5^ Emphasis is placed on both prophylactic and therapeutic dosed anticoagulation.^5^

The current anticoagulant of choice in the prevention of VTE amongst hospitalised COVID-19 infected patients is low molecular weight heparin (LMWH). Monitoring of LMWH has been evaluated through assessing anti-factor Xa (anti-Xa), through a measure of factor Xa enzyme (Xa) inhibition.^6^ Anti-Xa assessment has been found to improve the dosage regulation of LMWH in the treatment and prevention of VTE amongst SARS-CoV2 infected patients.^7,8,9^ A study by Trunfio et al.^10^ revealed that prompt correction of LMWH dosages based on an initial anti-Xa level measurement reduced SARS-CoV-2 related mortality.

In addition to the current gold standard of anti-Xa levels, current tests for monitoring LMWH include activated partial thromboplastin time and thrombin generation.^11^ Anti-Xa assays are not without limitations. Importantly, these assays are not widely available and often need to be referred to reference laboratories, which prolongs turnaround time, reducing their clinical utility. Drawbacks with anti-Xa level monitoring include the additional cost of testing with estimates as high as 55 United States dollars (USD; approximately 500 South African rand [ZAR] – 1000 ZAR, or approximately 27 USD – 55 USD) per patient per test.^6^ Pre-analytic factors which may impair the validity of anti-Xa levels include poor blood sampling technique, delays of two or more hours in sample analysis, inadequate centrifugation, as well as haemolysis. Repeated tests may be required to establish a trend in anti-Xa levels, further increasing costs. Biologic limitations include antithrombin deficiency, increased binding, and inactivation of heparin-binding proteins (as a result of infection and inflammation), obesity (linked to inadequate levels of LMWH because of the increased volume of distribution) and impaired renal function (decreased elimination of LMWH).^12^ The prolonged turnaround time required to obtain a result from overburdened laboratories is problematic. The meticulous timing that each test needs to adhere to may be challenging in many settings.^10^

Thromboelastography is a point-of-care non-invasive in vivo test carried out in a specialised machine known as a thromboelastograph.^12^ Thromboelastograms identify and measure dynamic changes in coagulation, identifying where abnormalities in coagulopathy are found. Thromboelastogram parameters consist of a reaction time (R-time), kinetics time (K-time), alpha angle, maximum amplitude (MA), and lysis at 30 min (A30).^12^ This is performed at the point of care, limiting the delay expected in laboratory-processed testing.^13^ There may be overall cost benefits to using thromboelastograms.^14^

According to a retrospective study of 32 SARS-CoV-2 infected patients, thromboelastogram analysis has proven to be useful for both screening for hypercoagulability and VTE, and also for determining appropriate dosing of anticoagulation therapy.^15^ A systematic review published by the International Society of Thrombosis and Haemostasis, assessing 153 articles, including 841 patients, revealed a good thromboelastogram detection rate of hypercoagulability associated with SARS-CoV-2.^16^

Currently, there is a paucity of data exploring the relationship between thromboelastograms, anti-Xa levels and LMWH. Furthermore, there has yet to be defined a specified time period after the administration of LMWH, when thromboelastography is to be done.^17^ Owing to the potential advantages of thromboelastograms which may address some limitations of anti-Xa testing, this study aimed to assess the correlation between thromboelastograms and D-dimers, and thromobelastograms and anti-Xa levels. The primary objective was to determine if there is an association between thromboelastogram coagulation index (TCI) and anti-Xa levels among COVID-19 positive patients. Secondary outcomes were to determine if there was a correlation between anti-Xa levels and thromboelastogram parameters, namely the R-time, K-time, and alpha angle.

## Methods

### Ethical considerations

Ethical clearance was obtained from the University of the Witwatersrand Human Research Ethics Committee (reference number: M230570). Written consent was obtained from each participant when collecting data directly from patients and adding to a database for the study. Measures taken to ensure confidentiality included obtaining informed consent, anonymising data, strict access controls to data, secure storage of data on the REDCap® electronic data capture system, clear confidentiality policies for all researchers involved and the signing of a confidentiality agreement. The necessary ethical clearance documentation has been provided.

### Setting

The study was a retrospective cross-sectional observational study at Chris Hani Baragwanath Academic Hospital COVID-19 and intensive care unit wards, between November 2020 and January 2021. Chris Hani Baragwanath Academic Hospital is a tertiary hospital located in Johannesburg, Gauteng, South Africa.

### Patient inclusion and exclusion criteria

Patients included in the study had reverse transcription polymerase chain reaction-confirmed SARS-CoV-2 infection, were ≥ 18 years old, and had severe SARS-CoV-2 disease. Severe disease was defined as oxygen saturation < 93% with a respiratory rate ≥ 25 breaths per min, requiring supplemental oxygen support without the need for invasive or non-invasive ventilation. Critical illness was defined as hypoxaemia and the need for additional ventilatory support, in the form of non-invasive or invasive ventilation.

Exclusion criteria for patients were: a prolonged activated partial thromboplastin time of > 50 s; patients receiving chronic anticoagulation therapy (including, but not limited to, aspirin, warfarin, clopidogrel, LMWH such as enoxaparin and direct-acting oral anticoagulants such as rivaroxiban); patients with prior VTE, currently receiving quinine or a derivative thereof; known thrombotic thrombocytopenia purpura or thrombotic thrombocytopenia purpura-like disease; known haemoglobinopathies; pregnancy; oestrogen replacement therapy; and patients not receiving anticoagulation acutely or having any contraindication to anticoagulation.

### Sample size

To achieve a correlation coefficient of 0.5 with 80% confidence and precision of approximately 10% between thromboelastograms and anti-Xa levels, as shown previously by Tekkesin et al.,^18^ a sample size of 40 was required. We collected an additional 5% (2 samples) to allow for test failures, thereby arriving at 42 samples in total.

### Data collection

Data collection was carried out by the primary investigator and supervisors. A database was created as part of a PhD for one of the supervisors, with data being collected from 01 November 2020 to 31 January 2021. The data were collected by both the primary investigator and the supervisors during this period and entered directly into the REDCap® electronic data capture tool.^19^ The data were then exported into Statistica® version 13.3 (TIBCO Software Inc., Santa Clara, California, United States) for data interpretation. Data extraction from this database was conducted from 01 March 2021 to 31 May 2021. Sociodemographic data were collected directly from patients, including age, gender and ethnicity.

Whole venous blood samples were drawn directly from patients from the antecubital vein using a tourniquet and syringe for thromboelastogram, D-dimer, and anti-Xa. These blood samples were drawn on admission (prior to enoxaparin administration), 48 h post admission (3 h after enoxaparin administration); and at clinical resolution (defined as resolution of hypoxia) or day 10 of illness.

Thromboelastograms were processed within 5 min of whole blood being taken, and the corresponding sample was taken to the laboratory for anti-Xa testing within the specified 5 min as per manufacturer’s instructions. The TEG® 6s machine (Haemonetics®, Boston, Massachusetts, United States) was then used by filling a droplet of blood and a droplet of reagent into a cartridge which is then processed. The result was displayed and printed revealing an R-time, K-time, alpha angle, MA, and TCI. Only results from the standard kaolin thromboelastograms were considered.

Blood samples for D-dimer and anti-Xa tests were stored in citrate tubes (Becton-Dickinson, Oxford, UK). D-Dimer, and anti-Xa tests were done by the Chris Hani Baragwanath Academic Hospital National Health Laboratory Service Haematology Department, accredited by the South African National Accreditation System (SANAS), ISO 15189. D-dimers were measured using the D-dimer PLUS assay (Siemens Healthcare Diagnostics Products GmbH, Marburg, Germany). The test requires a latex-agglutination test which is then processed using an automated quantitative turbidimetric D-dimer assay. Anti-Xa levels were measured using the STA®-Liquid Anti Xa assay (Diagnostica Stago PTY LTD, Doncaster, Australia). The recommended target reference ranges for adults on treatment is 0.6 IU/mL – 1.0 IU/mL, and 0.2 IU/mL – 0.6 IU/mL for prophylaxis.

Reaction time evaluates the time from coagulation cascade initiation to fibrin generation and clot propagation. It reflects activity of the coagulation cascade. A shorter R-time reflects hypercoagulability. It is calculated from the beginning of the test to the first detectable clot formation (2 mm). The normal reference range is 5 min – 10 min.^20^

Kinetics time determines the rate at which a clot is formed and is measured from the beginning of clotting to the proper formation of a clot (20 mm). It measures fibrin deposition and cross-linking. The normal reference range is 1 min – 2 min.^20^

The alpha angle determines the speed of clot growth and strengthening. It is the angle between R-time and a line from the time of clotting initiation to the point of maximal clot formation speed. The normal angle is 53°–72°. When decreased, it suggests a deficiency in fibrinogen.^20,21^

Maximum amplitude refers to the maximal amplitude of the thromboelastograph curve; the normal range is 50 mm – 70 mm.^20^

Lysis at 30 min depicts the speed of fibrinolysis and shows the percentage of reduction 30 min after MA. The normal range is 0% – 8%.^20^

The TCI assesses overall coagulation status, and the formula (Equation 1) is as follows:


CI=(−0.2454×R-time)+(0.0184×K-time)+(0.1655×MA)−(0.0241×alpha angle)−5.0220 [Eqn 1]

The normal TCI range is within −3.0 and +3.0 (3 standard deviations from the mean of zero). Hypercoagulability is a TCI greater than +3.0, and coagulopathy is a TCI less than −3.0.^20^

### Statistical analysis

Data were collected and managed using REDCap®^19^ electronic data capture tools hosted at the University of the Witwatersrand, and statistical analyses was performed using StatSoft, Inc. data analysis system, Statistica version 13.3 (www.statsoft.com; TIBCO Software Inc., Santa Clara, California, United States).Spearman’s correlation was used to determine if there was an association between anticoagulation test parameters. Student’s *t*-test and Mann Whitney *U* test were used to determine the relationship between the dosing groups of enoxaparin and coagulation tests.

A multiple linear regression model was performed (Online Supplementary Table 1) using the thromboelastogram parameters (R-time, K-time, alpha angle, MA and A30) to predict anti-Xa levels (Table 1). Only R-time, K-time and A30 were included in the final model, as *p*-values were ≤ 0.2 (a 20% probability that the correlation was a result of chance was considered acceptable). Alpha angle was omitted, as this is dependent on K-time. Only R-time was significantly associated with anti-Xa level, with a *p*-value of < 0.05.

**TABLE 1:** Regression summary of thromboelastogram results and anti-Xa levels of patients at 48 h post admission (*N* = 42), Chris Hani Baragwanath Hospital, Johannesburg, Gauteng, South Africa, 01 November 2020 to 31 January 2021.

Variable	Contribution strength (*b*†)	Contribution precision (s.e. of *b*†)	Direct change (*b*)	Change precision (s.e. of *b*)	*p*
Intercept	-	-	0.329	0.136	0.022
R-time	0.408	0.194	0.029	0.014	0.045
K-time	−0.047	0.202	−0.017	0.077	0.818
Lysis 30 (%)	−0.221	0.176	−0.098	0.078	0.218

Note: *R* = 0.47271293 *R*^2^ = 0.22345751 Adjusted *R*^2^ = 0.14025653 *F*[3.28] = 2.6858 *p*.

*b*, contribution strength; s.e., standard error; R-time, reaction time; K-time, kinetics time.

† , precision contribution and strength contribution.

## Results

### Baseline characteristics

**FIGURE 1:**
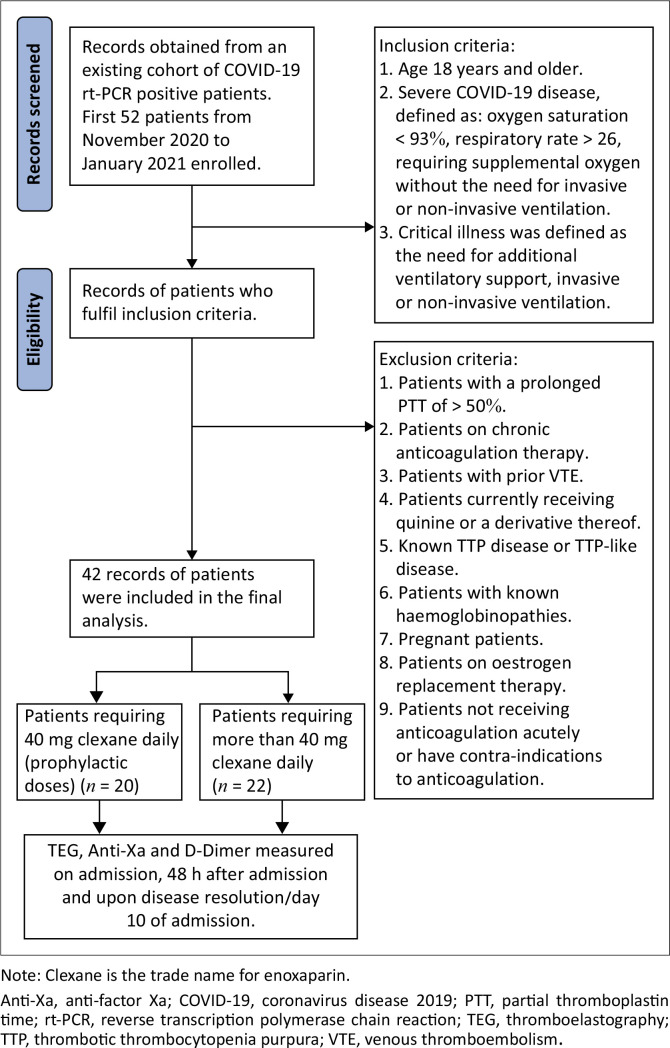
Study flow diagram of records reviewed of patients enrolled into the study between 01 November 2020 and 31 January 2021 at Chris Hani Baragwanath Hospital, Johannesburg, Gauteng, South Africa.

Forty-two participants were enrolled into the study (Figure 1). A total of 22 patients required a prophylactic dose (≤ 40 mg/day) of enoxaparin (52.38%), while 20 patients were treated with a therapeutic dose (> 40 mg/day) of enoxaparin (47.61%) (Table 2). Fifteen men and 27 women, with ages ranging from 33 years to 74 years, met the entry and exit criteria, enrolling them into the study. Patients with prior VTE, bleeding disorders, or on any anticoagulants, were excluded from the study to ensure a population which would not be biased towards bleeding or thrombosis.

**TABLE 2:** Baseline patient characteristics (*N* = 42).

Variable	All				Enoxaparin 40 mg daily (*n* = 22)		Enoxaparin > 40 mg daily (*n* = 20)	
	Median	IQR	*n*	%	*n*	%	*n*	%
**Sociodemographic factors**								
**Sex**								
Male	-	-	15	35.71	8	53.33	7	46.67
Female	-	-	27	64.28	12	44.44	15	55.56
**Age (years)**	61	49.5–67.5	42	100.00	-	-	-	-
**Ethnicity**								
Black African	-	-	37	88.09	16	43.24	21	56.76
Mixed-race	-	-	3	7.14	1	33.33	2	66.67
Asian	-	-	2	4.70	0	0.00	2	100.00
**Clinical factors**								
Sequential Organ Failure Assessment score	3	2–4	42	100.00	-	-	-	-
Overweight (defined as BMI > 30)	-	-	16	38.09	6	37.50	10	62.50
Patients with comorbid disease	-	-	31	73.80	12	38.71	19	61.29
Hypertensive	-	-	22	52.38	9	40.90	13	59.10
Diabetic	-	-	17	40.47	5	29.41	12	70.59
HIV positive	-	-	6	14.28	3	50.00	3	50.00
Renal failure	-	-	8	19.04	1	12.50	7	87.50
**Hospitalisation**								
Critical illness (ICU)	-	-	22	52.38	6	27.27	16	72.73
Hospital mortality	-	-	18	42.85	3	16.67	15	83.33
Discharged home alive	-	-	22	52.38	15	68.18	7	31.82
Step down to facility for oxygen therapy	-	-	1	1.92	0	0.00	1	100.00
Refused hospital therapy > 48 h post admission	-	-	1	1.92	1	100.00	0	0.00

IQR, interquartile range; BMI, body mass index; ICU, intensive care unit.

### Anti-Xa levels and thromboelastogram coagulation index

**FIGURE 2:**
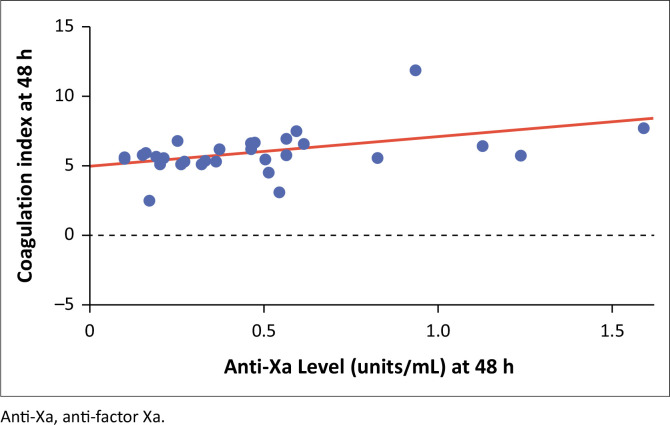
Correlation between anti-factor Xa and thromboelastogram coagulation index of the 42 patients enrolled between 01 November 2020 and 31 January 2021 at Chris Hani Baragwanath Hospital, Johannesburg, Gauteng, South Africa.

There was a statistically significant low to moderate correlation between anti-Xa levels and TCI, using Spearman’s correlation = 0.43 (*p* = 0.014) among patients admitted for suspected COVID-19 pneumonia (Figure 2).

### Anti-Xa and thromboelastogram parameters

**FIGURE 3:**
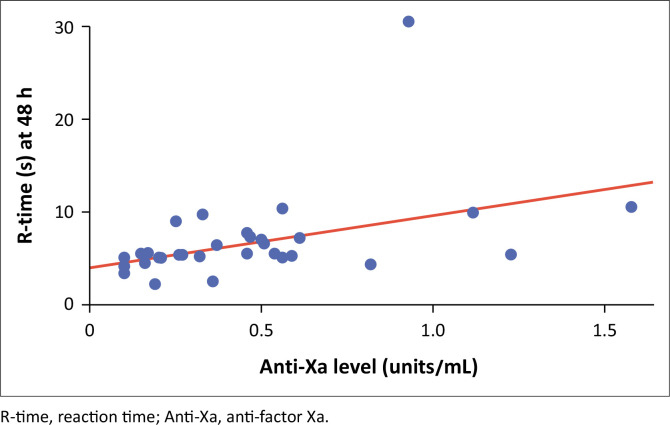
Correlation between anti-factor Xa and reaction time of the 42 patients enrolled between 01 November 2020 and 31 January 2021 at Chris Hani Baragwanath Hospital, Johannesburg, Gauteng, South Africa.

There was a statistically significant moderate correlation between anti-Xa levels and R-time, with Spearman’s correlation = 0.52, *p* = 0.002 (Figure 3).

**FIGURE 4:**
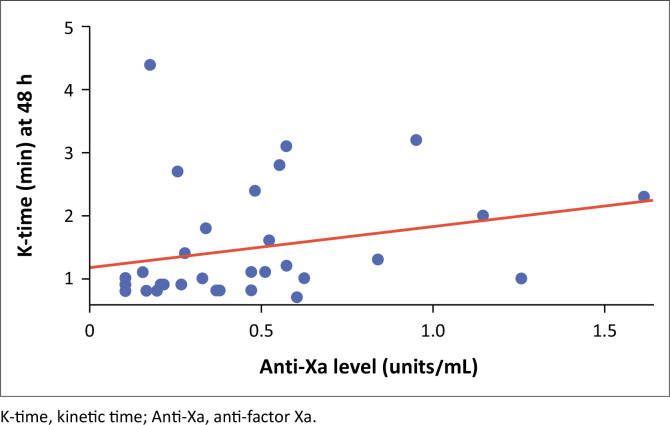
Correlation between anti-factor Xa and kinetics time of the 42 patients enrolled between 01 November 2020 to 31 January 2021 at Chris Hani Baragwanath Hospital, Johannesburg, Gauteng, South Africa.

There was a statistically significant low correlation between anti-Xa levels and K-time. with Spearman’s correlation = 0.35, *p* = 0.049 (Figure 4).

Although the K-time and alpha angle are mathematically correlated, we did not find a statistical correlation between anti-Xa levels and alpha angle.

There were also no significant correlations between D-dimer and thromboelastogram parameters.

Spearman Rank Order Correlations revealed no marked correlations between thromboelastogram and D-dimers, at a significance of *p* < 0.05.

### Enoxaparin dose, thromboelastogram parameters, and anti-Xa levels

Only anti-Xa levels were significantly associated with the dose of enoxaparin, while amongst the thromboelastogram parameters, lysis time at 30 min was significantly associated with the dose of enoxaparin (Online Supplementary Table 2).

## Discussion

The main finding of our study demonstrated an association between thromboelastogram parameters and anti-Xa levels 48 h after admission and anticoagulation with enoxaparin. Using TCI, we found that higher anti-Xa levels were associated with higher TCI values. This does not necessarily mean that TCI can be used as a surrogate marker for anti-Xa levels; it merely indicates that patients with higher thrombotic risk (higher TCI values) were correctly put onto therapeutic LMWH.

The mean TCI of our sample was 5.67 (5.14–6.27). This is significantly higher than the normal range of −3.0 to +3.0, indicating hypercoagulability. This is likely indicative of the hypercoagulability associated with COVID-19 and the need for LMWH. Tekkesin et al.,^18^ in a study conducted in Turkey in 2015, report all TCI values in their sample to be below 0.0 following administration of enoxaparin. This is a large discrepancy which could be explained by various factors, ranging from severity of illness of participants to enoxaparin preparations, and requires further investigation.

Tekkesin et al.^18^ found a correlation of 0.38 between TCI and anti-Xa levels 24 h post initiation of anticoagulation in Turkey. The TCI values, as expected, predictably dropped at higher anti-Xa levels. In our study, higher anti-Xa levels appear to either be associated with higher TCIs or there appears to be no association at all. Our study population comprised a group of patients with a higher pro-coagulable phenotype; patients were not excluded on the basis of comorbidities and organ dysfunction. Our data included subgroups with both prophylactic and therapeutic enoxaparin dosing. These differences may account for the variance in correlation between the two studies. Our study measured TCI and anti-Xa levels at 48 h post initial enoxaparin dosing.

Buckley et al., in the United States, 2021, looked at a similar population to ours (COVID-19), and also found a weak correlation (*r* = 0.30) between TCI and anti-Xa levels.^22^ The important differences in their study was that therapeutic anticoagulation was targeted using a continuous enoxaparin infusion for more than 24 h at the discretion of the treating physician, rather than radiologically confirmed VTE. No standardised anticoagulation protocol was used among different patients in the population, rather enoxaparin infusion rates were altered depending on anti-Xa levels. The differences in population characteristics may allude to the reasoning for our differences in results, such as: all patients in their study were admitted to the intensive care unit as opposed to 52% in our study, and almost 60% of their population was male compared to 35% in our data. We had a predominantly black African population, while less than 10% of their study population was black. Finally, median Sequential Organ Failure Assessment scores differed, with 5 versus 3 in our study data.^22^

We found a significant correlation between anti-Xa levels and R-time at 48 h, where higher anti-Xa levels appear to coincide with longer R-time. But this does not mean R-times can be used as a surrogate for anti-Xa levels. Tekkesin et al. found a correlation between anti-Xa levels and R-time, which was only significant at 4 h post first dose of anticoagulation and normalised by 12 h.^18^ This discrepant finding between our study and that of Tekkesin may be attributed to the underlying disease in our studies. Tekkesin et al. had a patient population who underwent surgical orthopaedic intervention, which, as postulated by Bunescu et al.^23^ in London, 2002, releases granular factor from platelets, which has an impact on reaction time. Granular factors are released during surgery but then end at or shortly after completion of surgical procedures. This may be one of the mechanisms of rapid R-time normalisation in the study done by Tekkesin et al.,^18^ as opposed to ongoing pro-coagulopathic processes in COVID-19. Klein et al.^17^ (United States, 2000) found a significant correlation between R-time and anti-Xa levels in a population of 24 patients undergoing orthopaedic surgery. Contrary to Tekkesin et al., the significance was maintained at both peak and trough levels, questioning the platelet granular factor theory.^17^

White et al. (Australia, 2011) found no correlation between anti-Xa levels and thromboelastogram R-time (*p* = 0.38).^24^ Their population of 50 patients were admitted to a coronary care unit and received therapeutic dosed enoxaparin (1 mg/kg twice daily); anti-Xa levels and thromboelastograms were taken after 48 h post first dose of anticoagulation. Of note, the non-significant correlation between anti-Xa levels and R-times may be attributed to sampling errors (mean time from dosing of enoxaparin to thromboelastogram measurement was 4.17 h ± 0.5 h), as this study failed to find a correlation between enoxaparin dose and anti-Xa levels. Despite all patients receiving therapeutic dosed enoxaparin, a cutoff of 100 mg/dose was implemented in patients weighing more than 100 kg in this study. White et al. had not factored in the decline in creatinine clearance as a result of age, which would impact on enoxaparin clearance.^24^

Buckley et al. found a weak but significant correlation between thromboelastogram R-time and anti-Xa level;^22^ however, the differences in this study compared to ours may be because of the nature of enoxaparin administration (continuous infusion vs daily and twice-daily dosing).

Artang et al. (Denmark, 2009) conducted a study using seven healthy male volunteers who were injected subcutaneously with dalteparin (LMWH) 120 IU/kg.^25^ Reaction time was then compared to anti-Xa levels at baseline, then at 2, 4, 5 and 24 h post dalteparin administration. At 24 h, a strong correlation of *r* = 0.82 was found. However, multiple differences are found in this study when compared to ours; most importantly, the LMWH used was not enoxaparin but rather dalteparin. Alongside this, the study consisted of seven male participants who were all healthy and did not suffer from the same disease process as our population (COVID-19).

### Thromboelastogram kinetics time and alpha angle

We found a statistically significant low correlation between anti-Xa levels and K-time (*r* = 0.35), which is different from the study by Buckley et al.,^22^ who found no relationship between anti-Xa levels and K-time (*r* = 0.08). This was likely because we measured the K-time at peak enoxaparin dose effect (3 h post dose) compared to the continuous infusion, which may not have achieved the optimal anti-Xa level. Furthermore, differences in our study population, especially regarding disease profile, may be contributory as previously mentioned.^22^

We failed to detect a statistically significant relationship between anti-Xa levels and MA, which is similar to the findings of Buckley et al.^22^ These findings are not surprising, as much as 80% of the MA is contributed to by platelet activity,^26^ which is not affected by enoxaparin.

Our study found no statistically significant correlations between D-dimer and thromboelastogram parameters. This has also been found by Chandel et al. in a study of 24 patients with severe COVID-19 pneumonia.^27^ Their study found a discordant or unpredictable relationship between clot burden and D-dimer levels. This may explain the lack of relationship between D-dimer, anti-Xa levels and thromboelastogram parameters.^27^

Although we found a statistically significant difference in A30 percentages between prophylactic and therapeutic dose enoxaparin, there was no clinically significant difference. This finding is confirmed by Lloyd-Donald et al. (Australia, 2020) who failed to find any clinically significant relationship in A30.^28^ However, our finding is an interesting one, and may require further investigation on the effect of enoxaparin on fibrinolysis.

### Limitations

Our study had several limitations which need to be considered. Firstly, we included a limited sample size, which is open to large variations in precision. Secondly, although we had two dosing strategies of enoxaparin, the exact dose in the therapeutic dosed group was not considered for the analysis. The data are also limited by use of a single LMWH medication (enoxaparin) and are likely not to apply exactly to other anticoagulants. The nature of a single centre study limits the generalisability of the findings. However, given the limited published data available, our study findings are not insignificant, given that they contribute the second largest published sample sized study.

### Conclusion

We have demonstrated that the TCI (comprising R-time, K-time, and alpha angle) is statistically significantly correlated with anti-Xa levels in this study sample receiving LMWH. In particular, TCI values appear to increase as anti-Xa levels increase. It would be useful to see if this finding is replicated in other studies and, if so, why. Our findings should be interpreted with reserve and do not mean that TCI can replace anti-Xa testing. Anti-Xa levels were associated with the dose of LMWH (as expected), with lysis time at 30 min being the thromboelastogram parameter associated with LMWH dose (an interesting finding worth further investigation regarding the relationship between LMWH and fibrinolysis).

The possibility should also be considered that the high TCI values obtained in patients with higher anti-Xa levels are merely a reflection of the hypercoagulability present in these patients (who are correctly, it would seem, treated with higher doses of enoxaparin), and that these high TCI values have no direct relationship with the measured anti-Xa levels at all, but rather with the particular patient group receiving higher enoxaparin doses.

This study serves as a basis for future research with larger sample sizes, including patients with varying disease profiles and severity of illness. Further studies should focus on threshold values for R-time and other thromboelastogram indices that indicate specific anticoagulation targets.

## References

[1] World Health Organization. Number of Covid-19 cases reported to WHO (cumulative total). 2021 [cited 2021 June 27]. Available from: https://data.who.int/dashboards/covid19/cases?n=c

[2] Poor HD. Pulmonary thrombosis and thromboembolism in COVID-19. Chest. 2021;160(4):1471–1480. 10.1016/j.chest.2021.06.016 34153340 PMC8213519

[3] Sakr Y, Giovini M, Leone M, et al. Pulmonary embolism in patients with coronavirus disease-2019 (COVID-19) pneumonia: A narrative review. Ann Intensive Care. 2020;10(1):124. 10.1186/s13613-020-00741-0 32953201 PMC7492788

[4] Jing H, Wu X, Xiang M, Liu L, Novakovic VA, Shi J. Pathophysiological mechanisms of thrombosis in acute and long COVID-19. Front Immunol. 2022;13:992384. 10.3389/fimmu.2022.992384 36466841 PMC9709252

[5] Schulman S, Sholzberg M, Spyropoulos AC, Zarychanski R, Resnick HE, Bradbury CA, et al. ISTH guidelines for antithrombotic treatment in COVID-19. JTH. 2022 Oct;20(10):2214–2225. 35906716 10.1111/jth.15808 PMC9349907

[6] Dutt T, Simcox D, Downey C, et al. Thromboprophylaxis in COVID-19: Anti-FXa – The missing factor? Am J Respir Crit Care Med. 2020;202(3):455–457. 10.1164/rccm.202005-1654le 32510975 PMC7397804

[7] Middeldorp S, Coppens M, Van Haaps TF, et al. Incidence of venous thromboembolism in hospitalized patients with COVID-19. J Thromb Haemost. 2020;18(8):1995–2002. 10.1111/jth.14888 32369666 PMC7497052

[8] Helms J, Tacquard C, Severac F, et al. High risk of thrombosis in patients with severe SARS-CoV-2 infection: A multicenter prospective cohort study. Intensive Care Med. 2020;46(6):1089–1098. 10.1007/s00134-020-06062-x 32367170 PMC7197634

[9] Klok FA, Kruip MJHA, Van Der Meer NJM, et al. Confirmation of the high cumulative incidence of thrombotic complications in critically ill ICU patients with COVID-19: An updated analysis. Thromb Res. 2020;191:148–150. 10.1016/j.thromres.2020.04.041 32381264 PMC7192101

[10] Trunfio M, Salvador E, Cabodi D, et al. Anti-Xa monitoring improves low-molecular-weight heparin effectiveness in patients with SARS-CoV-2 infection. Thromb Res. 2020;196:432–434. 10.1016/j.thromres.2020.09.039 33049598 PMC7543686

[11] Thomas O, Lybeck E, Strandberg K, Tynngård N, Schött U. Monitoring low molecular weight heparins at therapeutic levels: Dose-responses of, and correlations and differences between aPTT, anti-factor Xa and thrombin generation assays. PLoS One. 2015;10(1):e0116835. 10.1371/journal.pone.0116835 25625201 PMC4308107

[12] Jones A, Barnard S, Monis G. Anti-factor Xa for monitoring of unfractionated heparin therapy [homepage on the Internet]. UC Davis Health – Pathology and Laboratory Medicine. UC DAVIS HEALTH. 2017 [cited 2021 Oct 03]. Available from: https://health.ucdavis.edu/blog/lab-best-practice/anti-factor-xa-for-monitoring-of-unfractionated-heparin-therapy/2017/10

[13] Shaydakov ME, Sigmon DF, Blebea J. Thromboelastography [homepage on the Internet]. NCBI. 2021 [cited 2021 Oct 03]. Available from: http://www.ncbi.nlm.nih.gov/books/NBK537061/

[14] Jackson GNB, Ashpole KJ, Yentis SM. The TEG^®^ vs the ROTEM^®^ thromboelastography/thromboelastometry systems. Anaesthesia. 2009;64(2):212–215. 10.1111/j.1365-2044.2008.05752.x 19143701

[15] Collett LW, Gluck S, Strickland RM, Reddi BJ. Evaluation of coagulation status using viscoelastic testing in intensive care patients with coronavirus disease 2019 (COVID-19): An observational point prevalence cohort study. Aust Crit Care. 2021;34(2):155–159. 10.1016/j.aucc.2020.07.003 32773357 PMC7373052

[16] Kazi S, Noureldin A, Deng Y, Othman M. TEG/ROTEM: A tool to aid in the diagnosis and management of COVID 19 coagulopathies – A systematic review [homepage on the Internet]. ISTH. 2021 [cited 2021 Oct 03];PB0199. Available from: https://abstracts.isth.org/abstract/teg-rotem-a-tool-to-aid-in-the-diagnosis-and-management-of-covid-19-coagulopathies-a-systematic-review/

[17] Klein SM, Slaughter TF, Vail PT, et al. Thromboelastography as a perioperative measure of anticoagulation resulting from low molecular weight heparin: A comparison with anti-Xa concentrations. Anesth Analg. 2000;91(5):1091–1095. 10.1097/00000539-200011000-00009 11049889

[18] Tekkesin N, Tekkesin M, Kaso G. Thromboelastography for the monitoring of the antithrombotic effect of low-molecular-weight heparin after major orthopedic surgery. Anatol J Cardiol. 2015;15(11):932–937. 10.5152/akd.2014.5723 26574762 PMC5336946

[19] Harris PA, Taylor R, Minor BL, Elliott V, Fernandez M, O’Neal L, et al. The REDCap consortium: Building an international community of software platform partners. J Biomed Inform. 2019 Jul;95:103208. 31078660 10.1016/j.jbi.2019.103208 PMC7254481

[20] Whitton TP, Healy WJ. Review of thromboelastography (TEG): Medical and surgical applications. Ther Adv Pulm Crit Care Med. 2023;18:29768675231208426. 10.1177/29768675231208426 38107072 PMC10725099

[21] Shaydakov ME, Sigmon DF, Blebea J. Thromboelastography [homepage on the Internet]. Treasure Island: StatPearls Publishing; 2025 [cited 2025 Jan 21]. Available from: http://www.ncbi.nlm.nih.gov/books/NBK537061/30725746

[22] Buckley MS, Benanti GE, Gilbert B, Meckel J, Dzierba AL, MacLaren R. Correlation between heparin anti-Xa activity and thromboelastography in adult critically ill COVID-19 patients. Pharmacotherapy. 2023;43(8):795–803. 10.1002/phar.2829 37199139

[23] Bunescu A, Widman J, Lenkei R, Menyes P, Levin K, Egberg N. Increases in circulating levels of monocyte-platelet and neutrophil-platelet complexes following hip arthroplasty. Clin Sci (Lond). 2002;102(3):279–286. 10.1042/cs1020279 11869168

[24] White H, Sosnowski K, Bird R, Jones M, Solano C. The utility of thromboelastography in monitoring low molecular weight heparin therapy in the coronary care unit. Blood Coagul Fibrinolysis. 2012;23(4):304–310. 10.1097/MBC.0b013e32835274c0 22473047

[25] Artang R, Frandsen NJ, Nielsen JD. Application of basic and composite thrombelastography parameters in monitoring of the antithrombotic effect of the low molecular weight heparin dalteparin: An in vivo study. Thromb J. 2009;7:14. 10.1186/1477-9560-7-14 19903343 PMC2777119

[26] Harr JN, Moore EE, Chin TL, et al. Platelets are dominant contributors to hypercoagulability after injury. J Trauma Acute Care Surg. 2013;74(3):756–762; discussion 762–765. 10.1097/TA.0b013e3182826d7e 23425732 PMC3736746

[27] Chandel A, Patolia S, Looby M, Bade N, Khangoora V, King CS. Association of D-dimer and fibrinogen with hypercoagulability in COVID-19 requiring extracorporeal membrane oxygenation. J Intensive Care Med. 2021;36(6):689–695. 10.1177/0885066621997039 33641491 PMC8145413

[28] Lloyd-Donald P, Vasudevan A, Angus P. Comparison of thromboelastography and conventional coagulation tests in patients with severe liver disease. Clin Appl Thromb Hemost. 2020;26:107602962092591. 10.1177/1076029620925915 PMC7427018 32496878

